# The predictive value of platelet-to-high density lipoprotein cholesterol ratio and white blood cell-to-mean platelet volume ratio for coronary artery disease risk in STEMI patients

**DOI:** 10.3389/fcvm.2026.1843566

**Published:** 2026-05-29

**Authors:** Yongxue Xie, Li Mao, Fangling Luo, Jianbin Pi, Qingjun He

**Affiliations:** 1 The Eighth Clinical Medical College of Guangzhou University of Chinese Medicine, Guangdong, China; 2 Foshan Hospital of Traditional Chinese Medicine, Guangdong, China

**Keywords:** coronary artery lesions, coronary artery severity, platelet-to-high-density lipoprotein cholesterol ratio, ST-segment elevation myocardial infarction, white blood cell count to mean platelet volume ratio

## Abstract

**Background:**

This study aimed to evaluate the prognostic value of the platelet count to high-density lipoprotein cholesterol ratio (PHR) and the white blood cell count to mean platelet volume ratio (WMR) in patients with acute ST-segment elevation myocardial infarction (STEMI).

**Methods:**

We enrolled 87 acute STEMI patients (STEMI group) and 100 healthy individuals (control group). The STEMI group was stratified by the number of coronary artery lesions (single-, double-, or multi-vessel) and by disease severity using the Gensini score (mild, moderate, severe). Clinical data were compared, and statistical analyses were performed.

**Results:**

(I) PHR and WMR levels were significantly higher in the STEMI group than in controls (*P* < 0.05). However, PHR was significantly lower in the multi-vessel group than in the double- and single-vessel groups (*P* < 0.05), and lower in moderate/severe groups than in the mild group (*P* < 0.05). (II) PHR showed a negative correlation with the number of lesions (*r* = −0.672, *P* < 0.001) and severity (*r* = −0.400, *P* < 0.001). (III) Multivariate analysis identified PHR as an independent risk factor for both acute STEMI (OR 1.069, 95% CI 1.069–1.143, *P* = 0.049) and for moderate-to-severe coronary lesions (OR 0.958, 95% CI 0.938–0.979, *P* < 0.001). (IV) On the ROC curve, PHR demonstrated moderate predictive value for the occurrence of STEMI, involvement of two or more diseased vessels, and moderate-to-severe coronary lesions, with AUCs of 0.756, 0.881, and 0.870, respectively (all *P* < 0.001). Meanwhile, WMR was found to be a useful predictor specifically for the occurrence of STEMI (AUC = 0.951, *P* < 0.001).

**Conclusions:**

A lower PHR is associated with a greater number and severity of coronary lesions in STEMI patients. PHR and WMR are predictive of STEMI occurrence, and PHR is an important predictor of the number and severity of diseased coronary vessels.

## 1 Introduction

Due to the prevalence of unhealthy lifestyles among Chinese residents closely associated with cardiovascular disease (CVD) and accelerated population aging, the incidence and mortality rates of CVD continue to rise. Cardiovascular disease remains the leading cause of death among both urban and rural residents (1). Ischemic heart disease (IHD) is one of the most common types of cardiovascular disease. Acute coronary syndrome (ACS) represents the most severe and life threatening form of IHD, with an estimated annual global incidence of approximately 7 million cases (2). Since the 2012 European Society of Cardiology Guidelines (ESC) (3), ACS is primarily classified into two types: ST-elevation myocardial infarction (STEMI) and non-elevation myocardial infarction (NSTEMI). Reports indicate that STEMI patients exhibit worse short-term outcomes compared to NSTEMI patients (4), underscoring the critical importance of early STEMI identification and timely intervention.

The most common pathogenesis of STEMI involves plaque rupture leading to coronary thrombosis, with other mechanisms including vascular endothelial injury, immune-inflammatory responses, and abnormal lipid metabolism (5). The platelet-to-high-density lipoprotein cholesterol ratio (PHR) has been identified as a key indicator of inflammation and hypercoagulability. Clinical evidence suggests these states correlate with STEMI development (6, 7). However, studies examining PHR's predictive value for STEMI severity and coronary lesion number remain limited. Existing research confirms that complete blood count (CBC) is a useful biomarker for predicting cardiovascular disease, including platelet count, mean platelet volume, and white blood cell count (8). Currently, white blood cell count and mean platelet volume are often examined as independent factors associated with STEMI. The ratio of white blood cell count to mean platelet volume (WMR) has been used as a predictor of thrombosis in cardiovascular and cerebrovascular disease (9). However, sufficient evidence regarding the relationship between WMR and STEMI remains lacking. Given this, the present study investigates the relationship between PHR and WMR with the number and severity of coronary lesions in patients with acute STEMI to clarify their assessment value.

## 2 Materials and methods

### 2.1 Study population

This retrospective cross-sectional study enrolled a total of 87 patients diagnosed with acute ST-segment elevation myocardial infarction (STEMI) at Foshan Hospital of Traditional Chinese Medicine between January 2019 and September 2024, and underwent complete coronary angiography. Based on coronary angiography results (10), the enrolled patients were categorized into three groups: single-vessel lesion group (32 cases), double-vessel lesion group (27 cases), and multi-vessel lesion group (28 cases). Patients were further categorized based on coronary lesion severity: mild group (Gensini ≤ 40 points, 34 cases), moderate group (40 points < Gensini < 80 points, 31 cases), high-score group (Gensini ≥ 80 points, 22 cases).

Sample size calculation: *post-hoc* power analysis was performed using G*Power software (version 3.1; Heinrich Heine University Düsseldorf, Germany). The primary hypotheses were based on the associations of PHR and WMR with the number of diseased coronary vessels and the severity of coronary artery disease, with the latter dichotomized according to the Gensini score (mild/moderate vs. severe). A two-sided test was applied with a significance level (*α*) set at 0.05. The expected effect size (|*ρ*|) was conservatively set at 0.20, representing a small-to-moderate correlation commonly observed in cardiovascular biomarker studies. With a total sample size of 187 participants (87 STEMI patients and 100 healthy controls), the achieved statistical power (1−β) was calculated to be 0.92. This value exceeds the conventional threshold of 0.80, indicating that the current sample size is adequate to support the subsequent statistical analyses.

Inclusion Criteria: (I) Meets the diagnostic criteria for ST-segment elevation myocardial infarction (STEMI) as outlined in the <2019 Chinese Society of Cardiology (CSC) guidelines for the diagnosis and management of patients with ST-segment elevation myocardial infarction> (11). (II) Clinical coronary angiography demonstrates coronary artery lesion extent and severity consistent with STEMI. (III) Age>18 years. (IV) Complete clinical documentation. (V) Stable vital signs and condition. (VI) The patient has provided informed consent for this study.

Exclusion Criteria: (I) History of coronary artery intervention. (II) Concurrent severe diseases affecting cardiac function, such as severe valvular heart disease, dilated cardiomyopathy, or refractory heart failure. (III) Concurrent severe dysfunction of vital organs, including the liver, kidney, or brain. (IV) History of cerebral embolism or cerebral hemorrhage within the past 3 months. (V) Concurrent malignant tumors. (VI) Concurrent severe infections, diseases, or hematological disorders.

### 2.2 Research methods

#### 2.2.1 Collection of general data

A total of 87 patients with acute STEMI admitted to Foshan Hospital of Traditional Chinese Medicine between January 2019 and September 2024 were selected as the STEMI group. Additionally, 100 healthy individuals undergoing routine physical examinations during the same period were selected as the control group (non-STEMI group) after clinical symptom assessment and exclusion of STEMI and other diseases based on imaging and laboratory indicators. General clinical data for both groups were collected and organized, including gender, BMI, age, history of hypertension, history of diabetes, history of long-term smoking, and history of heavy alcohol consumption.

#### 2.2.2 Laboratory data collection

Venous blood samples were collected from all patients in a fasting state prior to surgery and from all healthy individuals in a fasting state. Testing was performed by the Laboratory Department of Foshan Hospital of Traditional Chinese Medicine. The following parameters were collected: Platelet count (PLT), Platelet Distribution Width (PDW), Mean Platelet Volume (MPV), Platelet Crit (PCT), White Blood Cell Count (WBC), Serum Uric Acid (UA), High-Density Lipoprotein Cholesterol (HDL-C), Neutrophil Count (NEUT), Lymphocyte Count (LTMH), Monocyte Count (MONO), Low-Density Lipoprotein Cholesterol (LDL-C). Complete blood count parameters, including PLT, PDW, MPV, PCT, WBC, NEUT, LYM, and MONO, were measured using an automated hematology analyzer (XN-9000, Sysmex Corporation, Kobe, Japan). UA, HDL-C, and LDL-C were measured using an automated biochemical analyzer (AU5800, Beckman Coulter, Brea, CA, USA) with standard enzymatic methods. Using the collected data, calculate the platelet-to-high-density lipoprotein cholesterol ratio (PHR) and the white blood cell count-to- platelet volume ratio (WMR).

#### 2.2.3 Coronary angiography data collection

At least two cardiologists jointly analyzed coronary angiography results and assessed coronary artery lesions. Based on angiographic findings, the number of diseased coronary arteries in study subjects was evaluated, categorizing them into single-vessel lesion group, double-vessel lesion group, and multi-vessel lesion group. The Gensini score for coronary angiography was calculated and compared among subjects based on the number of diseased major coronary artery branches and the degree of stenosis. The Gensini score is calculated by multiplying the weight coefficient corresponding to each lesion site by the quantitative score corresponding to the degree of stenosis and summing the results (see Table 1) (12).

**Table 1:** Gensini scoring criteria.

Severity of stenosis	Score	Lesion site	Score
1%–25%	1	Left main trunk	5
26%–50%	2	Proximal segment of the left anterior descending artery, proximal segment of the circumflex artery	2.5
51%–75%	4	Left anterior descending artery, mid-segment	1.5
76%–90%	8	Distal segment of left anterior descending artery, middle segment of circumflex artery, distal segment of circumflex artery	1.0
91%–99%	16	Right coronary artery, diagonal branch, obtuse marginal branch, posterior descending artery	1.0
100%	32	Other branches	0.5

#### 2.2.4 Associated comorbidities

Baseline comorbidities were recorded and defined according to standard clinical criteria. Hypertension was defined as (13) systolic blood pressure (SBP) ≥140 mmHg and/or diastolic blood pressure (DBP) ≥90 mmHg on repeated measurements, or current use of antihypertensive medication. Diabetes mellitus was defined as (14) fasting plasma glucose ≥7.0 mmol/L, 2-hour plasma glucose ≥11.1 mmol/L during a 75 g oral glucose tolerance test, glycated hemoglobin ≥6.5%, or current glucose-lowering therapy. Dyslipidemia was defined as (15) low-density lipoprotein cholesterol (LDL-C) ≥3.4 mmol/L and/or high-density lipoprotein cholesterol (HDL-C) <1.0 mmol/L in males or <1.3 mmol/L in females, or current lipid-lowering medication. Long-term smoking was defined as (16) smoking ≥10 cigarettes per day for ≥1 year, and heavy alcohol consumption as daily alcohol intake ≥40 g for males or ≥20 g for females for ≥1 year (17).

#### 2.2.5 Medication for the associated comorbidities

According to the 2025 AHA/ACC/Multisociety Guideline for the Prevention, Detection, Evaluation, and Management of High Blood Pressure in Adults (13). The recommended first-line drug classes are: thiazide diuretics (e.g., hydrochlorothiazide), long-acting dihydropyridine calcium channel blockers (e.g., amlodipine), ACE inhibitors (ACEi) or angiotensin receptor blockers (ARBs).

According to the 2025 Focused Update of the 2019 ESC/EAS Guidelines for the Management of Dyslipidaemias (15), the cornerstone of pharmacotherapy for patients with dyslipidemia is statin therapy. For primary prevention, lipid-lowering drugs are recommended for very high-risk individuals with an LDL-C ≥ 70 mg/dL and for high-risk individuals with an LDL-C ≥ 100 mg/dL. If the LDL-C goal is not achieved with the maximum tolerated dose of a statin, combination therapy with ezetimibe is recommended as the next step. For patients who are intolerant to statins or have contraindications, bempedoic acid is now recommended with a Class I indication for both primary and secondary prevention.

According to the American Diabetes Association (ADA) Standards of Care in Diabetes-2026 (14), for patients with type 2 diabetes and established ASCVD (including STEMI), sodium-glucose cotransporter 2 (SGLT2) inhibitors or glucagon-like peptide-1 (GLP-1) receptor agonists with proven cardiovascular benefit are recommended as part of glucose-lowering therapy. Metformin remains a reasonable first-line agent for glucose management in patients with type 2 diabetes without established ASCVD or at lower cardiovascular risk.

### 2.3 Ascertainment of PHR

PHR was calculated using the complete blood count and lipid profile measurements. Peripheral venous blood samples were collected from all participants upon admission prior to interventional procedures. Platelet count (PLT) was measured using an automated hematology analyzer, and high-density lipoprotein cholesterol (HDL-C) levels were assessed using standard enzymatic methods. PHR was calculated as PHR = PLT (×10⁹/L)/HDL-C (mmol/L) (18). This formula has been widely adopted in recent cardiovascular studies as a novel indicator reflecting systemic inflammation and hypercoagulable states (19).

### 2.4 Ascertainment of WMR

WMR was derived from the complete blood count parameters. White blood cell (WBC) count and mean platelet volume (MPV) were measured using an automated hematology analyzer, as specified in the standard laboratory procedures. MPV is a parameter that reflects platelet size and has been recognized as an indicator of platelet activation and thrombotic potential. WMR was calculated as WMR = WBC (×10⁹/L)/MPV (fL) (20). WMR, as a novel composite inflammatory and thrombotic marker combining leukocyte count and platelet morphology, has recently been investigated as a predictor of long-term outcomes in myocardial infarction patients (21).

### 2.5 Observation indicators

(I) Compare the baseline characteristics between the STEMI group and the non-STEMI group, as well as among patients with different numbers of diseased coronary artery vessels. (II) Observe the levels of PHR and WMR in acute STEMI patients with varying numbers of diseased coronary artery vessels. (III) Examine the correlation between the number of diseased coronary artery vessels (and their severity) and the levels of PHR and WMR. (IV) Evaluate the potential of PHR and WMR levels in assessing the number of diseased coronary artery vessels and the severity of coronary artery lesions in acute STEMI patients.

### 2.6 Statistical analysis

Data processing and analysis were performed using SPSS 26.0 statistical software. Normality was assessed using the Shapiro–Wilk test (for groups with a sample size <50) and the Kolmogorov–Smirnov test (for groups with a sample size ≥50). For normally distributed quantitative data, results are presented as Mean ± SD. Differences between two groups were compared using an independent samples *t*-test. Differences among three groups with homogeneous variance were analyzed using one-way ANOVA, with pairwise comparisons performed using SNK-q tests. For nonnormally distributed data, results were expressed as *M* (P_25_, P_75_). Differences between two groups were assessed using the Mann–Whitney *U* test, while differences among three groups were evaluated using the Kruskal–Wallis rank sum test. Count data were presented as case numbers (%), with intergroup comparisons performed using chi-square tests. Spearman correlation analysis assessed the relationship between PHR and WMR with the number of coronary lesions. Univariate logistic regression analyzed predictors of STEMI and moderate-to-severe coronary lesions, while multivariate logistic regression identified independent risk factors for STEMI and moderate-to-severe coronary stenosis. The predictive value of the ratio was analyzed by plotting worker characteristics and ROC curves. *P* < 0.05 was considered statistically significant.

### 2.7 Ethics statement

The retrospective data used in this study were entirely sourced from Foshan Hospital of Traditional Chinese Medicine. The study protocol was reviewed by the Ethics Committee of Foshan Hospital of Traditional Chinese Medicine. As this is a retrospective study utilizing anonymized data, the committee granted an exemption from ethical review (Approval No: KY[2026]161) and all subjects in this study signed informed consent forms In accordance with the requirements of the ethics committee.

## 3 Results

### 3.1 Analysis of general clinical characteristics

General clinical characteristics of patients in the STEMI group and the non-STEMI group are shown in Table 2. The STEMI group had a higher proportion of males, long-term smokers, and patients with a history of hypertension. PCT, WBC, UA, NEUT, MONO, LDL-C, PHR, and WMR were significantly higher in the STEMI group compared to the non-STEMI group, while HDL-C was significantly lower (*P* < 0.05).

**Table 2:** Comparison of general characteristics and laboratory indicators between STEMI and non-STEMI groups [*n* (%)/mean ± SD].

Clinical data	STEMI group (*n* = 87)	Non-STEMI group (*n* = 100)	*Z*/*χ*^2^/*t*-value	*P*-value
Male	77 (88.5)	54 (54)	26.405	<0.001
BMI (kg/m^2^)	24.3 ± 3.1	24.0 ± 2.9	0.786	0.432
Long-term smoking history	41 (47.1)	20 (20)	15.576	<0.001
Hypertension history	42 (48.3)	33 (33)	4.520	0.034
Age (years)	55 (53, 56)	44 (46, 48)	−4.733	<0.001
Platelet count (PLT ×10^9^/L)	257.21 ± 62.05	245.61 ± 49.55	−1.416	0.158
Platelet volume distribution width (PDW %)	15.70 (13.76, 14.41)	15.90 (14.52, 15.09)	−1.573	0.116
Mean platelet volume (MPV fL)	9.90 (9.82, 10.10)	9.65 (9.70, 9.93)	−0.985	0.325
Platelet crit (PCT %)	0.25 (0.24, 0.26)	0.22 (0.21, 0.23)	−3.559	<0.001
White blood cell count (WBC ×10^9^/L)	11.73 (11.31, 12.15)	5.70 (5.57, 5.84)	−11.024	<0.001
Serum uric acid (UA μmol/L)	409.81 ± 95.77	318.30 ± 62.23	−6.057	<0.001
High-density lipoprotein cholesterol (HDL-C mmol/L)	1.11 (1.08, 1.14)	1.38 (1.37, 1.45)	−7.087	<0.001
PHR	239.85 ± 71.25	180.62 ± 54.71	−5.962	<0.001
WMR	1.20 ± 0.38	0.59 ± 0.13	−15.000	<0.001
Neutrophil count (NEUT ×10^9^/L)	8.92 ± 3.56	3.28 ± 0.91	−15.263	<0.001
Lymphocyte count (LYM×10^9^/L)	2.37 (2.17,2.58)	1.81 (1.80,1.92)	−0.691	0.490
Monocyte count (MONO ×10^9^/L)	0.58 (0.56,0.63)	0.36 (0.34,0.37)	−7.199	<0.001
Low-density lipoprotein cholesterol (LDL-C mmol/L)	3.66 (3.53,3.78)	3.01 (2.81,3.55)	−5.376	<0.001

<0.05, significant; *t*, independent samples *t*-test; *u*, Mann–Whitney *U* test; *c*, chi-square tests.

General clinical characteristics of patients in the single-vessel, double-vessel, and multi-vessel lesion groups are shown in Table 3. The multi-vessel lesion group had a higher proportion of patients with a history of hypertension. With lower PIT, PCT, and WMR compared to the single-vessel and double-vessel groups. HDL-C was higher in the multi-vessel and two-vessel lesion groups than in the single-vessel lesion group, while PHR was lower in the multi-vessel lesion group than in the single-vessel lesion group. All differences were statistically significant (*P* < 0.05).

**Table 3:** Comparison of general characteristics and laboratory indicators in STEMI groups with different numbers of coronary lesions [*n* (%)/mean ± SD/(P_25_, P_75_)].

Clinical data	Single-vessel disease group (*n* = 32)	Two-vessel disease group (*n* = 27)	Multi-vessel disease group (*n* = 28)	*χ*^2^/*F*/*H-*value	*P* value
Male	30 (93.8)	21 (77.8)	26 (92.9)	3.823	0.155
BMI (kg/m^2^)	24.5 ± 3.2	25.1 ± 3.5	25.4 ± 3.3	1.275	0.284
Hypertension history	9 (28.1)	15 (55.6)	18 (64.3)	8.651	0.013
Diabetes history	4 (12.5)	6 (22.2)	7 (25)	1.663	0.488
Long-term smoking history	15 (46.9)	11 (40.7)	10 (35.7)	0.773	0.681
Heavy drinking	5 (15.6)	3 (11.1)	4 (14.3)	0.332	0.927
Age (years)	50 ± 10.49	58.63 ± 12.45*	55.43 ± 10.09	4.666	0.012
Heart rate (HR beats/minute)	81 ± 15.44	82.48 ± 19.40	82.11 ± 11.43	0.041	0.109
Systolic blood pressure (SBP mmHg)	128.41 ± 18.12	123.26 ± 16.08	125.82 ± 17.59	0.647	0.526
Diastolic blood pressure (DBP mmHg)	81.25 ± 14.31	77.15 ± 15.23	78.43 ± 13.03	0.652	0.524
Platelet count (PLT ×10^9^/L)	283 (273.55, 299.14)	263 (247.15, 269.96)	213 (210.05, 235.16)**^▴^	15.630	<0.001
Platelet volume distribution (PDW %)	14.288 (13.75, 14.83)	13.86 (13.29, 14.43)	14.07 (13.46, 14.68)	0.359	0.836
Mean platelet volume (MPV fL)	9.70 (9.49, 9.90)	10 (9.64, 10.04)	10.38 (10.06, 10.69)	4.484	0.106
Platelet volume index (PVI %)	0.27 ± 0.55	0.25 ± 0.5	0.23 ± 0.04**^▴^	7.055	0.001
White blood cell count (WBC ×10^9^/L)	12.10 (11.96, 13.48)	11.70 (10.70, 12.23)	10.64 (10.26, 11.46)	3.419	0.181
Serum uric acid (UA μmol/L)	420.22 ± 92.72	389.82 ± 91.79	418.26 ± 103.21	0.868	0.424
High-density lipoprotein cholesterol (HDL-C mmol/L)	0.99 (0.94, 1.01)	1.2 (1.13, 1.21)**	1.18 (1.15, 1.25)**	18.489	<0.001
PHR	298.43 ± 64.87	224.41 ± 45.70**	187.78 ± 46.97**	33.036	<0.001
WMR	1.32 ± 0.41	1.18 ± 0.38	1.07 ± 0.32*	3.464	0.036
Neutrophil count (NEUT ×10^9^/L)	9.37 (8.55, 10.20)	8.00 (7.66, 9.33)	8.72 (8.14, 9.47)	0.565	0.754
Lymphocyte count (LYM ×10^9^/L)	2.65 (2.29, 3.01)	2.17 (1.88, 2.46)	2.25 (1.82, 2.68)	0.859	0.651
Monocyte count (MONO ×10^9^/L)	0.62 ± 0.25	0.63 ± 0.29	0.54 ± 0.26	1.613	0.446
Low-density lipoprotein cholesterol (LDL-C mmol/L)	3.57 ± 0.93	3.52 ± 1.10	3.89 ± 0.91	1.155	0.320

<0.05, significant; *F*, one-way ANOVA; *Q*, SNK-*q* tests; *H*, Kruskal–Wallis rank sum test; *c*, chi-square tests; *Q*-test, compared with the single-vessel lesion group *P < 0.05, **P < 0.01; compared with the two-vessel lesion group ^▴^*P* < 0.05.

Laboratory indicators relevant to this study for patients in the mild, moderate, and severe groups are shown in Table 4. PHR was lower in the moderate-severe group than in the mild group, and PCT was lower in the severe group than in the mild group, with all differences being statistically significant (*P* < 0.05).

**Table 4:** Laboratory indicators in groups with different degrees of coronary artery disease [*n* (%)/mean ± SD].

Clinical data	Mild group (*n* = 34)	Moderate group (*n* = 31)	Severe group (*n* = 22)	*χ*^2^/*F v*alue	*P* value
BMI (kg/m^2^)	24.6 ± 3.1	25.0 ± 3.4	25.3 ± 3.2	1.028	0.361
Hypertension history	15 (44.1)	14 (45.2)	13 (59.1)	1.428	0.490
Diabetes history	5 (14.7)	5 (16.1)	5 (22.7)	0.678	0.712
Dyslipidemia history	30 (88.2)	27 (87.1)	20 (90.9)	0.189	0.910
Platelet volume index (PVI %)	0.27 ± 0.06	0.25 ± 0.05	0.23 ± 0.05*	3.338	0.040
PHR	294.14 ± 64.77	205.99 ± 54.15**	203.64 ± 46.55**	25.426	<0.001
WMR	1.32 ± 0.38	1.10 ± 0.40*	1.14 ± 0.32	3.08	0.051

<0.05, significant; *F*, one-way ANOVA; *q*, SNK-*q* tests; *H*, Kruskal–Wallis rank sum test; *c*, chi-square tests; *Q*-test, compared with the single-vessel lesion group *P < 0.05, **P < 0.01.

The medication profile for comorbidities is presented in Table 5. This table systematically summarizes the use of medications targeting the three main comorbidities, namely hypertension, diabetes mellitus, and hyperlipidemia, in the study population (a total of 187 participants, including 87 patients with STEMI and 100 healthy controls). For each category of medication, the table shows the number and proportion of users among the total population, as well as among patients with hypertension (*n* = 75), diabetes mellitus (*n* = 32), and hyperlipidemia (*n* = 94).

**Table 5:** Pharmacological management of associated comorbidities in the study population [*n*, %)].

Associated comorbidities	Pharmacological management [*n*, %)]							
Hypertension	Bisoprolol (8, 10.7)	Metoprolol (17, 22.7)	Captopril (8, 10.7)	Azilsartan (2, 2.7)	Irbesartan (8, 10.7)	Levamlodipine (9, 12.0)	Nifedipine (4, 5.3)	Valsartan (9, 12.0)
Diabetes mellitus	Dapagliflozin (3, 9.4	Acarbose (17, 53.1)	Metformin (15, 46.9)	Glimepiride (2, 6.3)	Glipizide (1.3.1)			
Dyslipidemia	Rosuvastatin (39, 41.5)	Atorvastatin (43, 45.7)	Ezetimibe (1, 1.1)					

### 3.2 Correlation analysis between PHR and WMR ratios and the number of coronary lesion branches

**Figure 1:**
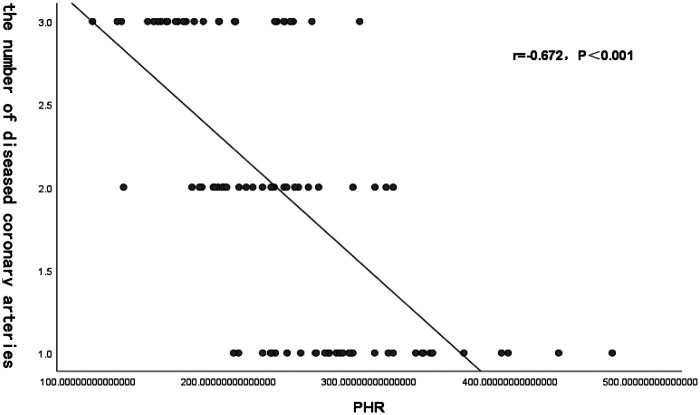
Correlation between PHR and the number of coronary artery lesions.

**Figure 2:**
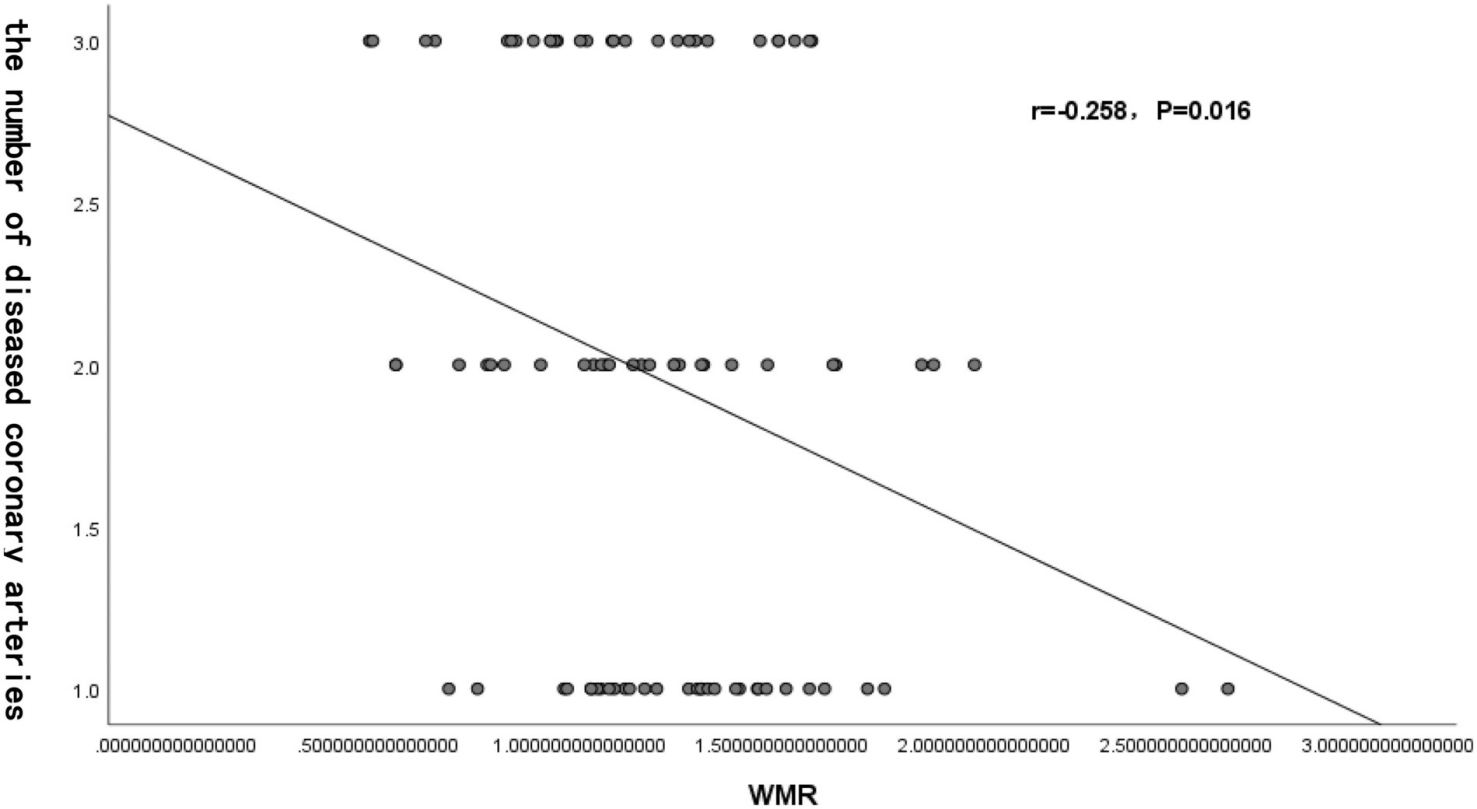
Correlation between WMR and the number of coronary artery lesions.

**Figure 3:**
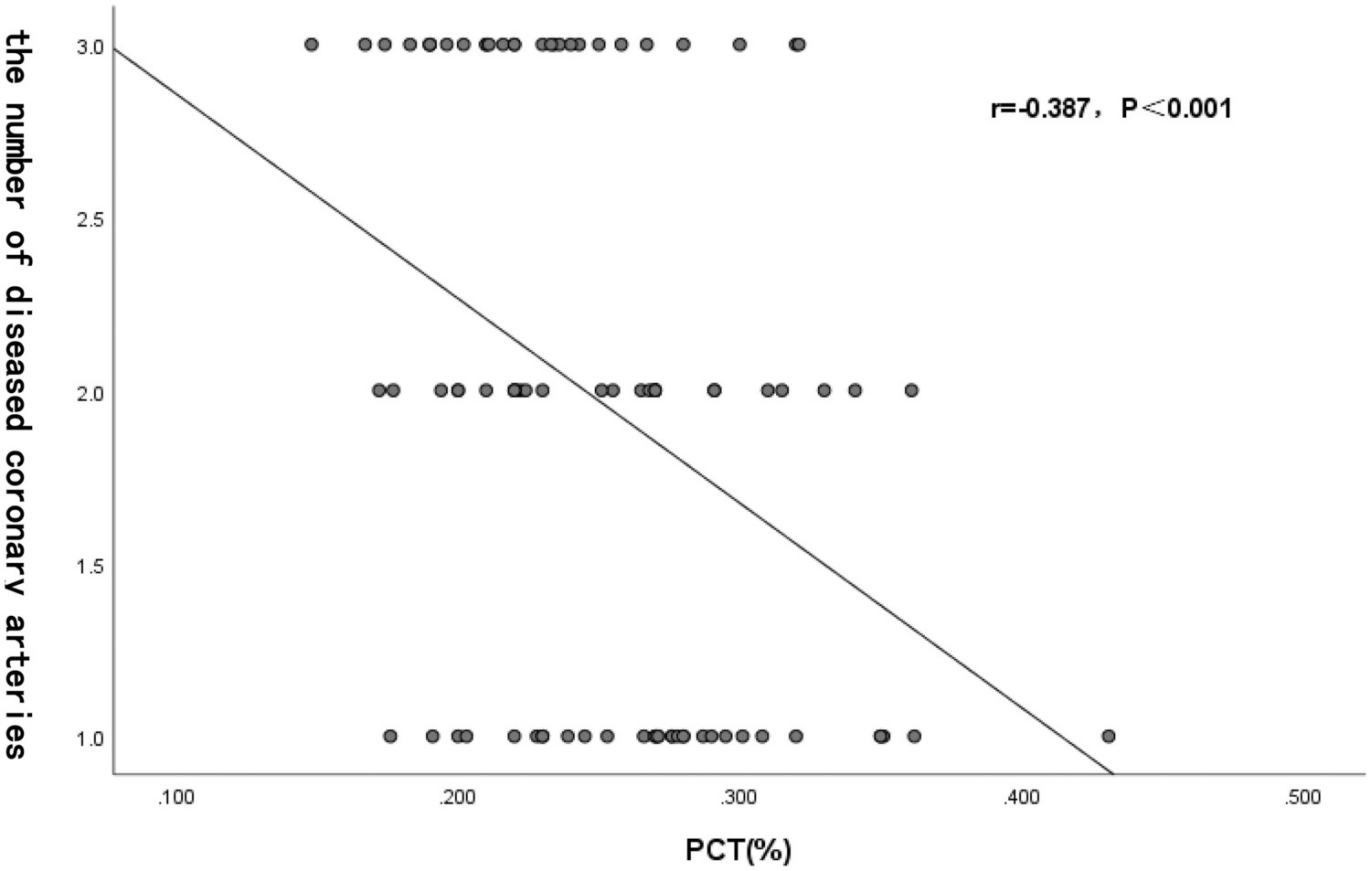
Correlation between PCT and the number of coronary artery lesions.

Spearman correlation analysis indicated a negative correlation between the PHR and the number of diseased coronary arteries (*r* = −0.672, *P* < 0.001) (see Figure 1). Similarly, the WMR showed a negative correlation with the number of diseased coronary arteries (*r* = −0.258, *P* = 0.016) (see Figure 2). Additionally, the PCT was also negatively correlated with the number of diseased coronary arteries (*r* = −0.387, *P* < 0.001) (see Figure 3).

### 3.3 Correlation analysis between PHR and Gensini score

**Figure 4:**
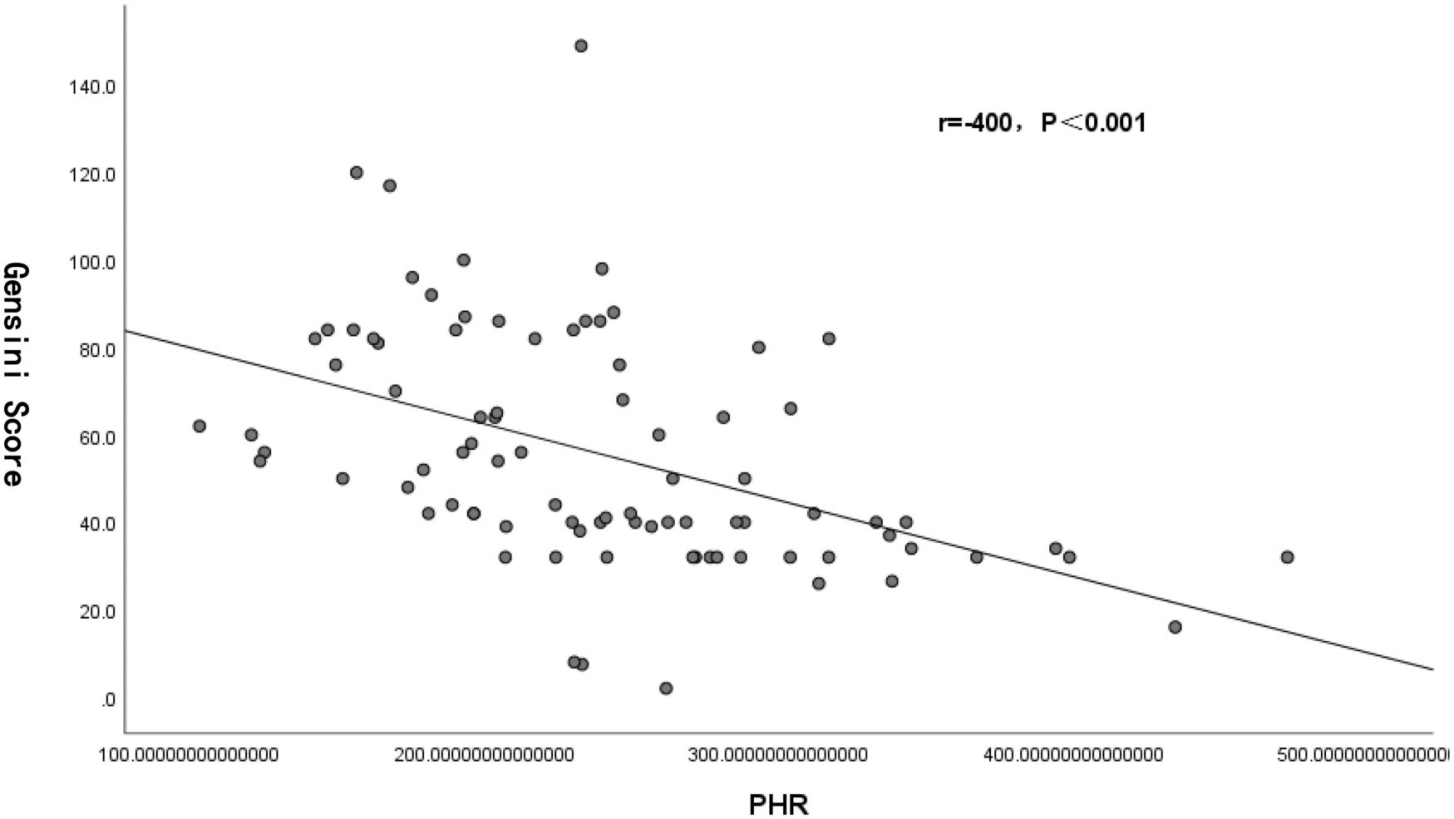
Correlation between PHR and the Gensini score.

Spearman correlation analysis indicated that PHR negatively correlated with coronary lesion severity (Gensini score) (*r* = −0.400, *P* < 0.001) (see Figure 4).

### 3.4 Risk factor analysis in acute STEMI patients

Factors with *P* < 0.05 in both the STEMI group and non-STEMI group were included in univariate logistic regression analysis. Results indicated that male gender, long-term smoking history, hypertension history, age, WBC, UA, HDL-C, NEUT, PHR, and WMR were predictive factors for acute STEMI. Factors showing statistically significant differences in univariate logistic regression analysis (*P* < 0.05) were included in multivariate logistic regression analysis. Due to linear correlations among PCT, WBC, HDL-C, PHR, and WMR, the final model included male gender, long-term smoking history, history of hypertension, age, serum uric acid, PHR, WMR, neutrophil count, and monocyte count were included in the multivariate logistic regression analysis. Results showed that the OR for PHR was 1.069, with a 95% CI ranging from 1.069 to 1.143, and *P* = 0.049, indicating that PHR is an independent risk factor for acute STEMI (see Table 6).

**Table 6:** Logistic regression analysis in acute STEMI patients.

Variable	Univariate analysis			Multivariate analysis		
	OR	95% CI	*P*	OR	95% CI	*P*
Male	6.559	3.045–14.128	<0.001			
Long-term smoking history	0.240	0.120–0.484	<0.001			
Hypertension history	0.526	0.280–0.987	0.045			
Age (year)	1.069	1.037–1.102	<0.001			
White blood cell count (WBC ×10^9^/L)	3.952	2.603–6.002	<0.001			
Serum uric acid (UA μmol/L)	1.015	1.010–1.020	<0.001			
High-density lipoprotein cholesterol (HDL-C mmol/L)	0.003	0.000–0.024	<0.001			
PHR	1.016	1.010–1.022	<0.001	1.069	1.069–1.143	0.049
WMR	0.002	0.000–0.012	<0.001			
Neutrophil count (NUET ×10^9^/L)	5.307	3.077–9.155	<0.001			
Low-density lipoprotein cholesterol (LDL-C mmol/L)	1.284	0.917–1.798	0.146			

### 3.5 Analysis of risk factors for moderate-to-severe coronary artery lesions

Factors with *P* < 0.05 in the STEMI group with varying numbers of coronary artery lesions were included in univariate logistic regression analysis. Results indicated that a history of hypertension, age, PLT, PCT, WBC, HDL-C, PHR, and WMR were predictive factors for moderate-to severe coronary artery lesions. Factors showing statistically significant differences in univariate logistic regression analysis (*P* < 0.05) were included in multivariate logistic regression analysis. Due to linear correlations among platelet volume, white blood cell count, high-density lipoprotein cholesterol, PHR, and WMR, the final multivariate model included history of hypertension, age, platelet count, platelet volume, PHR, and WMR. Results showed that the OR for PHR was 0.958, with a 95% CI of 0.938–0.979 (*P* < 0.001), indicating that PHR is an independent risk factor for moderate-to severe coronary artery disease (see Table 7).

**Table 7:** Logistic regression.

Variable	Univariate analysis			Multivariate analysis		
	OR	95% CI	*P*	OR	95% CI	*P*
Hypertension history	0.273	0.109–0.686	0.006			
Age (years)	1.071	1.023–1.121	0.003			
Platelet count (PLT ×10^9^/L)	0.988	0.980–0.996	0.005			
Platelet volume index (PVI %)	0.002	0.001–0.216	0.021			
White blood cell count (WBC ×10^9^/L)	0.868	0.754–0.999	0.049			
High-density lipoprotein cholesterol (HDL-C mmol/L)	7.008	3.499–18.229	<0.001			
PHR	0.970	0.958–0.983	<0.001	0.958	0.938–0.979	<0.001
WMR	12.214	1.823–81.812	0.010			

### 3.6 PHR and WMR in assessing STEMI occurrence, number of lesions in two or more vessels, and moderate-to-severe coronary artery lesions

**Figure 5:**
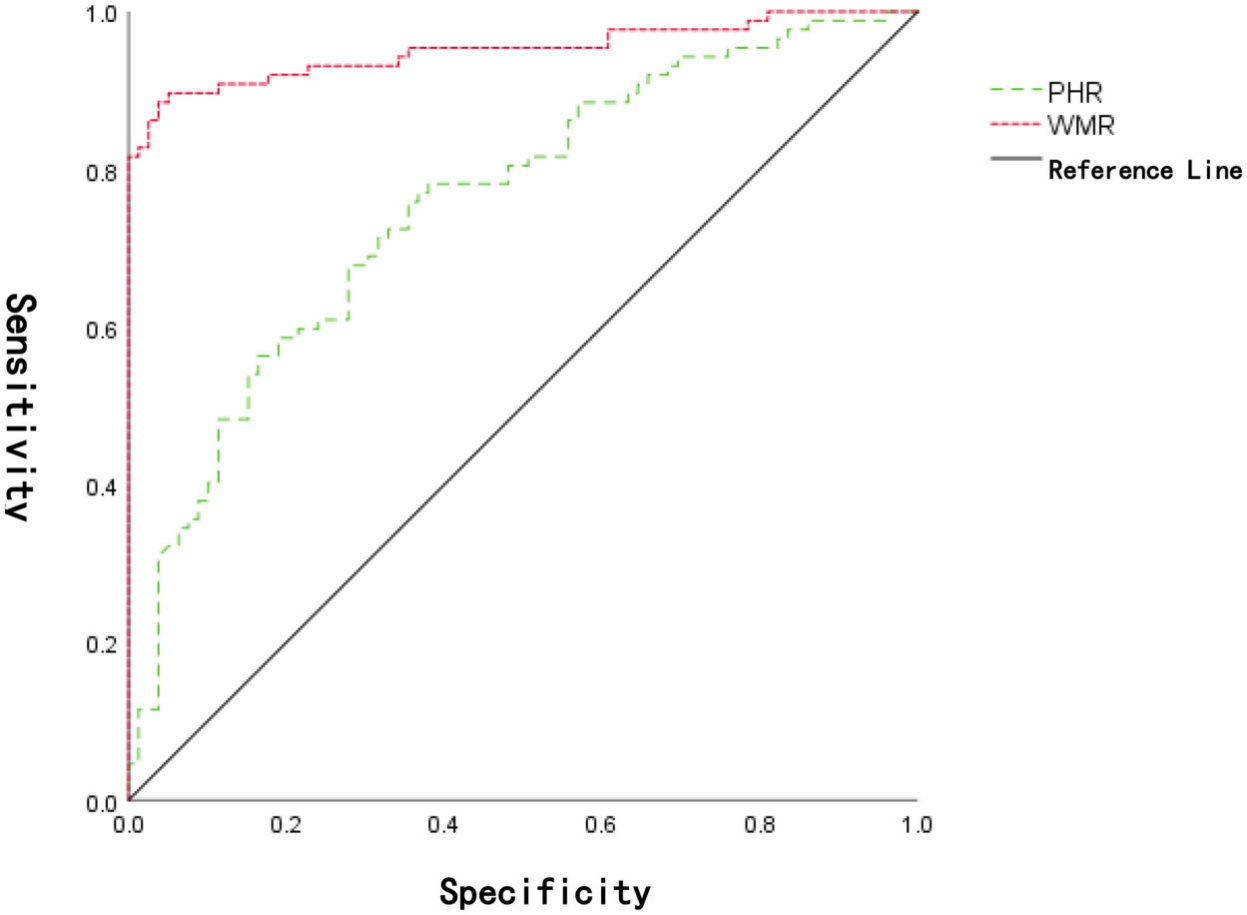
ROC curves for PHR and WMR in predicting STEMI occurrence.

The predictive performance of PHR and WMR for STEMI occurrence was evaluated using ROC curves. Results showed that the area under the curve (AUC) for PHR was 0.756 (95% CI: 0.683–0.829, *P* < 0.001). The cutoff value of 191.796 yielded the highest predictive value for STEMI occurrence, with specificity and sensitivity of 0.646 and 0.759 respectively. The area under the curve (AUC) for WMR was 0.951 (95% CI: 0.917–0.986, *P* < 0.001). The cutoff value of 0.778 yielded the highest predictive value for STEMI occurrence, with specificity and sensitivity of 0.962 and 0.885 respectively (see Figure 5).

**Figure 6:**
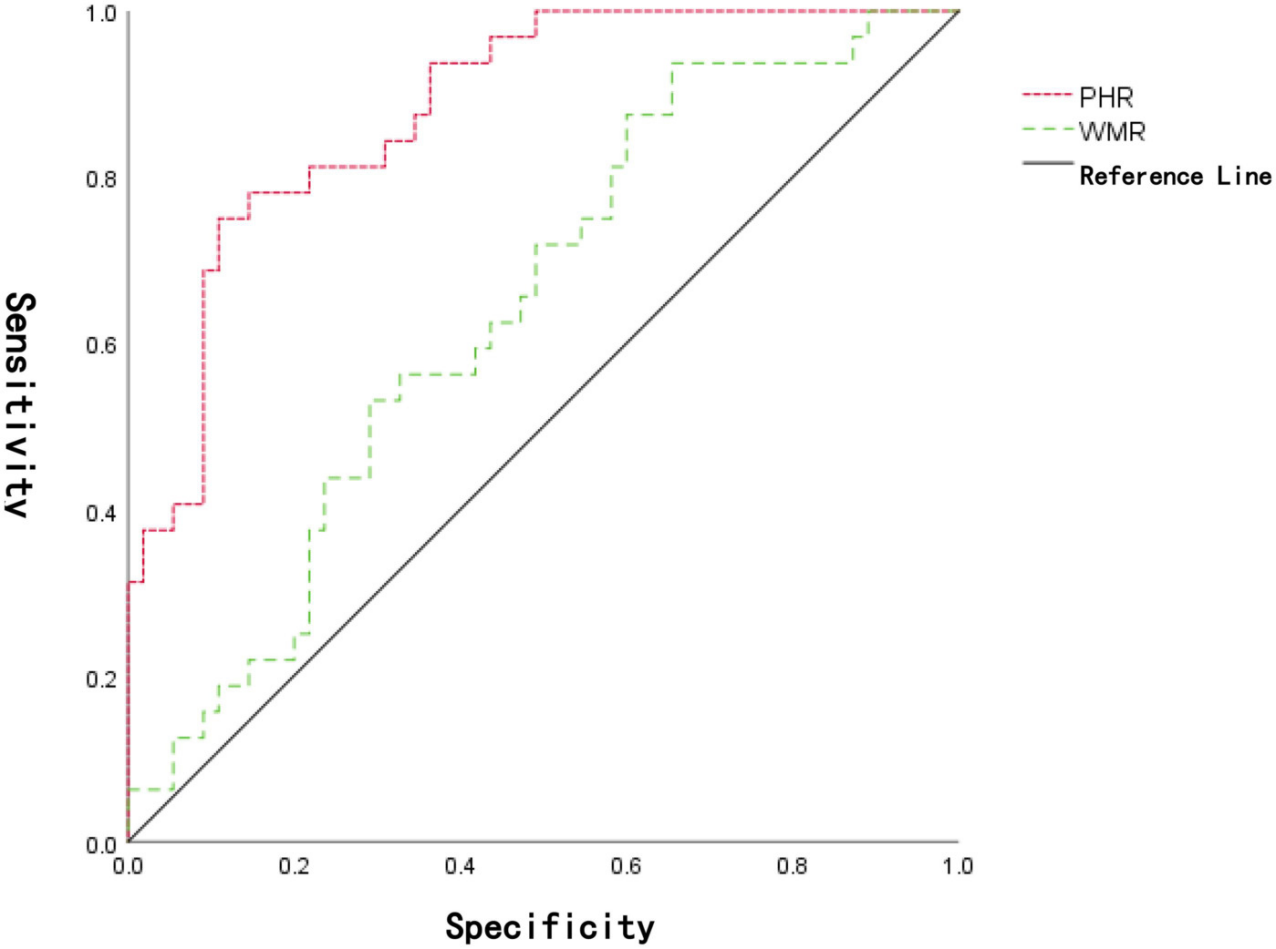
ROC curves of PHR and WMR for predicting the presence of two or more diseased vessels in STEMI.

ROC curves were used to evaluate the predictive performance of PHR and WMR for the number of lesions in STEMI patients with two or more affected vessels. Results showed that the area under the curve (AUC) for PHR was 0.881 (95% CI: 0.812–0.951, *P* < 0.001). At a cutoff value of 260.373, the predictive value for STEM with two or more lesion branches was highest with specificity and sensitivity of 0.891 and 0.750 respectively. The area under the WMR curve (AUC) was 0.641 (95% CI: 0.524–0.758, *P* = 0.029). At a cutoff value of 0.778, the predictive value for SEMI occurrence was highest, with specificity and sensitivity of 0.345 and 0.938 respectively (see Figure 6).

**Figure 7:**
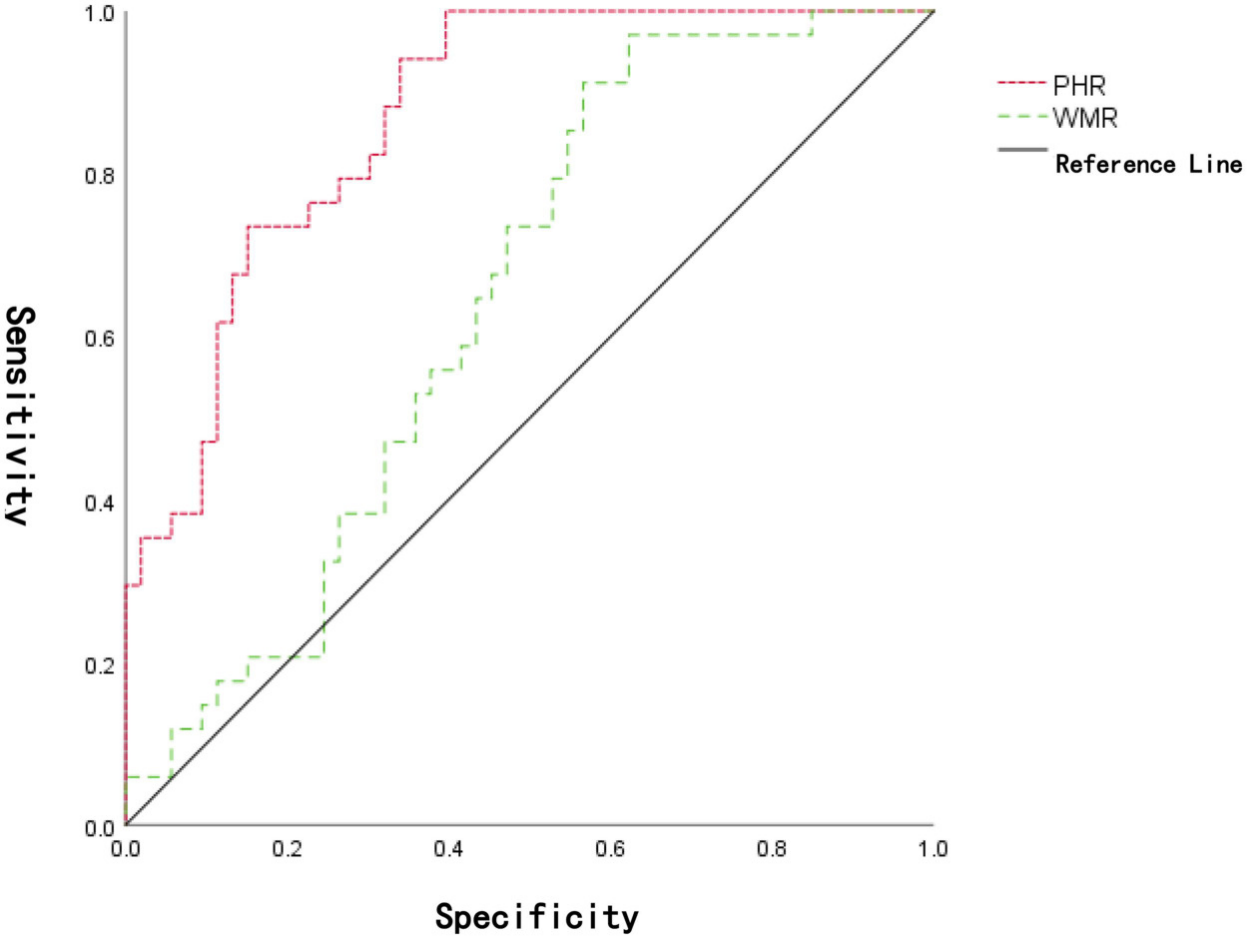
ROC curves of PHR and WMR for predicting moderate-to-severe lesions in STEMI.

The predictive performance of PHR and WMR for moderate-to-severe coronary lesions in STEMI was evaluated using ROC curves. Results showed that the area under the curve (AUC) for PHR was 0.870 (95% CI: 0.799–0.941, *P* < 0.001). The cutoff value of 205.881 yielded the highest predictive value for moderate-to-severe coronary lesions in STEM, with specificity and sensitivity of 0.604 and 1.000 respectively (see Figure 7). The area under the WMR curve (AUC) were 0.644 (95% CI: 0.530–0.759, *P* = 0.024). The cutoff value of 0.95 L yielded the highest predictive value for STEMI occurrence, with specificity and sensitivity of 0.377 and 0.971 respectively (see Figure 7).

## 4 Discussion

This study focuses on patients with STEMI, the most severe type of acute coronary syndrome (ACS). This population accounts for approximately 30% of all ACS cases and represents a major cause of cardiovascular death worldwide (22). Although percutaneous coronary intervention (PCI) has significantly improved the immediate prognosis of these patients, the improvement in clinical outcomes has plateaued in recent years. Some patients experience poor myocardial repair and long-term heart failure due to ischemia-reperfusion injury (IRI) and coronary microvascular obstruction (MVO) (23). These pathological conditions are reflected in this study by elevated PHR and WMR. Notably, patients in the early stage of STEMI still retain some physiological compensatory capacity and have not yet progressed to acute hemodynamic deterioration, which provides a critical time window for clinical intervention. By integrating convenient indicators such as PHR and WMR for early risk assessment, targeted interventions can be implemented before compensatory mechanisms fail.

In the analysis of general clinical characteristics, the STEMI group had higher PHR than the non-STEMI group (*P* < 0.001), and PHR showed good predictive value in distinguishing STEMI patients from non-STEMI subjects (AUC = 0.756). This finding may be attributed to an imbalance between the enhanced prothrombotic effect of platelets and the reduced vascular protective function mediated by HDL-C (24). Platelets play a central role in the pathophysiology of coronary artery disease (CAD), and a high platelet count may promote a prothrombotic state. Several underlying mechanisms have been identified. One study (25) found that the distribution of platelet subpopulations was significantly correlated with angiographic findings, and the procoagulant platelet subpopulation was markedly upregulated in patients with coronary artery lesions. The study proposed that a high platelet count accompanied by an increased proportion of procoagulant subpopulations could significantly enhance local thrombin generation and accelerate fibrin network formation, ultimately leading to a denser and more stable thrombus burden. Von Hundelshausen et al. (26) found that platelet-derived chemokines exert pro-atherosclerotic effects. Activated platelets release various chemokines, such as C-X-C motif chemokine ligand 4 (CXCL4) and C-C motif chemokine ligand 5 (CCL5), which are deposited on the endothelium and promote the recruitment of immune cells to the vessel wall, thereby directly participating in the inflammatory process of atherosclerosis. Studies have confirmed that the aggregation of platelets and neutrophils is a key step in the exacerbation of inflammatory response during IRI (27). Activated platelets bind to P-selectin glycoprotein ligand 1 (PSGL-1) on the leukocyte surface via P-selectin, forming stable platelet-leukocyte aggregates (PLAs). These aggregates not only directly block microvessels but also promote the recruitment and infiltration of leukocytes into the infarcted myocardium, thereby increasing the thrombus burden. Low levels of HDL-C may be associated with impaired reverse cholesterol transport and reduced anti-inflammatory and antioxidant capacities (28). Relevant studies have shown that low HDL-C is associated with the occurrence of STEMI and higher in-hospital and one-year mortality rates (29). LDL-C transports lipid molecules from the liver and deposits them in peripheral tissues or central blood vessels, and excessive LDL-C can lead to occlusion of arteries supplying major organs. The most core anti-atherosclerotic function of HDL-C is to promote cholesterol efflux capacity (CEC), which removes excess cholesterol from macrophages in the arterial wall and transports it to the liver for metabolism. Research has shown that the cholesterol efflux capacity of HDL-C and its subfractions (HDL2b, 2a, 3a, 3b, 3c) is significantly reduced in patients with STEMI (*P* < 0.01) (30). Meanwhile, another study found that cholesterol efflux capacity is negatively correlated with the inflammatory marker Interleukin-1β (IL-1β) (31). Therefore, STEMI patients with both impaired CEC and high IL-1β levels can be identified as a very high-risk subgroup for recurrent cardiovascular events, suggesting that HDL-related anti-inflammatory dysfunction contributes to adverse outcomes in STEMI.

However, it was surprisingly found that multivariate analysis in this study revealed a significant negative correlation between PHR and the number of diseased coronary vessels (OR = 1.069, 95% CI: 1.069–1.143, *P* = 0.049) as well as the severity of coronary artery disease (OR = 0.958, 95% CI: 0.938–0.979, *P* < 0.001). This trend may be attributed to extensive peripheral platelet consumption due to severe coronary thrombosis and relative changes in HDL-C levels. As early as 2000, a study (32) found that platelet counts were significantly lower in the acute myocardial infarction (AMI) group compared with the control group (*P* < 0.001). This finding suggests that platelets may be extensively consumed during acute coronary events. A study published in Hebei Medicine in 2025 (33) further confirmed that platelet counts were lower in the severe coronary artery disease group than in the mild-to-moderate disease group, and Spearman correlation analysis showed a negative correlation between platelet count and the severity of coronary artery disease (*r* = −0.397, *P* < 0.05). In patients with severe coronary artery disease, a large number of platelets may be activated or recruited to atherosclerotic plaques and infarcted myocardial areas, leading to a relative decrease in platelet count in peripheral blood. Compared with a single indicator such as platelet count, combined indicators are now often used to predict disease onset and progression. PHR is a marker reflecting inflammation and hypercoagulability status in whole blood. Recent studies have further revealed the potential predictive value of PHR in various cardiovascular diseases, including stroke and cardiovascular mortality (34). Jialal et al. (35) suggested that PHR may be a biomarker for the risk of atherothrombosis. A retrospective study of 1,317 patients with ACS published in 2026 (36) found that PHR had a U-shaped association with 1-year all-cause mortality, with both low and high levels associated with an increased risk of death. This finding provides some support for the present study, in that the very low PHR group (Q1) had a significantly increased risk of death (aHR = 2.077, *P* = 0.006), suggesting a poor prognosis associated with a severe consumption state. The study indicated that platelet consumption may not only result from severe thrombosis but may also be related to inflammation-mediated consumption, as activated platelets may be cleared more rapidly by the immune system at inflammatory sites, leading to a relative decrease in platelet count in peripheral blood. The U-shaped curve of PHR suggests the existence of an optimal window (cutoff point 213.60), which provides a new quantitative tool for risk stratification in future STEMI patients. Currently, most studies focus on the poor prognosis associated with high PHR, whereas the negative correlation found in this study offers a new clinical perspective: in the acute phase of STEMI, low PHR may primarily reflect a severe thrombotic burden rather than an ideal metabolic protective state. To confirm the above mechanistic hypotheses, further investigations should aim to directly assess the relationship between PHR and microvascular obstruction (MVO), as measured by the extent of MVO on myocardial contrast echocardiography (MCE) and the area of MVO on cardiac magnetic resonance (CMR).

In the analysis of general clinical characteristics, the STEMI group had higher WMR than the non-STEMI group (*P* < 0.001), and WMR showed good predictive value in distinguishing STEMI patients from non-STEMI subjects (AUC = 0.951). This finding may be attributed to an imbalance between inflammatory burden and platelet reactivity. Inflammatory markers have gradually become a major focus in predicting the progression and prognosis of cardiovascular diseases. WBC has been extensively confirmed by research to be associated with cardiovascular diseases, including coronary artery disease, and serves as one of the inflammatory markers for predicting disease progression and prognosis (37). Leukocytes exert direct damaging effects during myocardial ischemia-reperfusion injury and activate systemic inflammatory responses, thereby promoting plaque instability and thrombosis (38). Mean platelet volume (MPV) is a potential marker of platelet activation. Highly active and larger platelets are important determinants of the thrombotic process leading to complete coronary occlusion and subsequent STEMI. However, during the acute phase of STEMI, when a large number of platelets are consumed in the peripheral blood, MPV may also decrease, resulting in an elevated WMR. Weng et al. (39) found that the reduction in the proportion of larger platelets during inflammation may be due to the synthesis of procoagulant and proinflammatory factors and the release of platelets stored in the spleen, while these platelets are rapidly recruited to inflammatory sites, activated, and destroyed, leading to a decrease in MPV in patients with inflammation.

The most important finding of our study is that the predictive performance of WMR for the occurrence of STEMI (AUC = 0.951) is significantly better than its ability to differentiate the severity of coronary artery lesions. The SYNTAX score is an anatomical scoring system based on coronary angiography results and is used to quantify the complexity of coronary artery lesions. However, Demir et al. (21) found no significant correlation between WMR and the SYNTAX score in patients with NSTEMI (*P* = 0.14). Therefore, we speculate that WMR primarily reflects acute systemic inflammation and platelet activation status rather than the anatomical burden of atherosclerosis. This suggests that WMR may reflect not the complexity of coronary arteries but rather inflammation and platelet activation. Meanwhile, studies have also confirmed that WMR can serve as an important predictor of long-term mortality and major adverse cardiovascular events (MACE) in patients with non-ST-segment elevation myocardial infarction, without affecting the severity of coronary artery disease (21). Therefore, WMR has greater advantages as an early warning indicator for the occurrence of STEMI, but other indicators need to be combined for further risk stratification.

Our study has several limitations that should be considered. First, the sample size is limited (*n* = 187), and this is a single-center study, which limits the generalizability of the conclusions. Second, we only used baseline complete blood count results and did not assess changes in hematological parameters during follow-up. Third, we did not measure other procoagulant or proinflammatory markers, such as coagulation factors, D-dimer, fibrin monomer, C-reactive protein, interleukin-6 and interleukin-8, as well as oxidative stress parameters that may be associated with PHR and WMR. These factors may have introduced confounding effects on the results. Future studies should integrate multidimensional clinical data and novel biomarkers to develop more accurate risk prediction models for coronary artery disease.

## 5 Conclusion

In summary, compared to healthy individuals, STEMI patients exhibit elevated PHR and WMR levels. As PHR levels decrease, the number of coronary artery lesion branches in STEMI patients increases, and the severity of coronary artery stenosis also worsens. These findings collectively suggest that treatment strategies for acute STEMI patients should prioritize controlling hypercoagulability and systemic inflammation. The application of PHR as an indicator may assist in evaluating coronary artery lesions in STEMI patients and identifying high-risk individuals at an early stage, thereby providing objective evidence for clinical decision-making.

## Data Availability

The raw data supporting the conclusions of this article will be made available by the authors, without undue reservation.

## References

[1] The Writing Committee of the Report on Cardiovascular Health and Diseases in China. Report on cardiovascular health and diseases in China 2022: an updated summary. Biomed Environ Sci. (2023) 36(8):669–701. 10.3967/bes2023.106 37711081

[2] Bhatt DL Lopes RD Harrington RA. Diagnosis and treatment of acute coronary syndromes: a review. J Am Med Assoc. (2022) 327(7):662–75. 10.1001/jama.2022.0358 35166796

[3] Thygesen K Alpert JS Jaffe AS Simoons ML Chaitman BR White HD. Third universal definition of myocardial infarction. Glob Heart. (2012) 7(4):275–95. 10.1016/j.gheart.2012.08.001 25689940

[4] Bouisset F Ruidavets JB Dallongeville J Moitry M Montaye M Biasch K. Comparison of short- and long-term prognosis between ST-elevation and non-ST-elevation myocardial infarction. J Clin Med. (2021) 10(2):180. 10.3390/jcm10020180 33430516 PMC7826729

[5] Harrington DH Stueben F Lenahan CM. ST-elevation myocardial infarction and non-ST-elevation myocardial infarction: medical and surgical interventions. Crit Care Nurs Clin North Am. (2019) 31(1):49–64. 10.1016/j.cnc.2018.10.002 30736935

[6] Alyamani M Campbell S Navarese E Welsh RC Bainey KR. Safety and efficacy of intracoronary thrombolysis as adjunctive therapy to primary PCI in STEMI: a systematic review and meta-analysis. Can J Cardiol. (2021) 37(2):339–46. 10.1016/j.cjca.2020.03.034 32739451

[7] Granger CB Kochar A. Understanding and targeting inflammation in acute myocardial infarction: an elusive goal. J Am Coll Cardiol. (2018) 72(2):199–201. 10.1016/j.jacc.2018.05.006 29976294

[8] Seo IH Lee YJ. Usefulness of complete blood count (CBC) to assess cardiovascular and metabolic diseases in clinical settings: a comprehensive literature review. Biomedicines. (2022) 10(11):68. 10.3390/biomedicines10112697 PMC9687310 36359216

[9] Guetl K Raggam RB Muster V Gressenberger P Vujic J Avian A. The white blood cell count to mean platelet volume ratio for the prediction of chronic limb-threatening ischemia in lower extremity artery disease. J Clin Med. (2019) 8(10):1593. 10.3390/jcm8101593 31581728 PMC6832925

[10] Liu Y Ye T Chen L Jin T Sheng Y Wu G. Systemic immune-inflammation index predicts the severity of coronary stenosis in patients with coronary heart disease. Coron Artery Dis. (2021) 32(8):715–20. 10.1097/MCA.0000000000001037 33826540

[11] Chinese Society of Cardiology of Chinese Medical Association, Editorial Board of Chinese Journal of Cardiology. 2019 Chinese society of cardiology (CSC) guidelines for the diagnosis and management of patients with ST-segment elevation myocardial infarction. Chin J Cardiol. (2019) 47(10):766–83. 10.3760/cma.j.issn.0253-3758.2019.10.003 31648459

[12] Gensini GG. A more meaningful scoring system for determining the severity of coronary heart disease. Am J Cardiol. (1983) 51(3):606. 10.1016/S0002-9149(83)80105-2 6823874

[13] Kanbay M Copur S Sarafidis P Ferro CJ Ortiz A. 2025 AHA/ACC/AANP/AAPA/ABC/ACCP/ACPM/AGS/AMA/ASPC/NMA/PCNA/SGIM guideline for the prevention, detection, evaluation and management of high blood pressure in adults: a commentary from the European renal best practice (ERBP). Nephrol Dial Transplant. (2026):gfag055. 10.1093/ndt/gfag055 41805831 PMC13624793

[14] American Diabetes Association Professional Practice Committee for Diabetes. 2. Diagnosis and classification of diabetes: standards of care in diabetes-2026. Diabetes Care. (2026) 49(1):S27–49. 10.2337/dc26-S002 41358893 PMC12690183

[15] Mach F Koskinas KC Roeters Van Lennep JE Tokgözoğlu L Badimon L Baigent C. 2025 Focused update of the 2019 ESC/EAS guidelines for the management of dyslipidaemias. Eur Heart J. (2025) 46(42):4359–78. 10.1093/eurheartj/ehaf190 40878289

[16] World Health Organization. WHO report on the global tobacco epidemic, 2025: warning about the dangers of tobacco (2025).

[17] National Academies of Sciences, Engineering, and Medicine, Health and Medicine Division, Food and Nutrition Board, Committee on Review of Evidence on Alcohol and Health. Review of Evidence on Alcohol and Health. Washington, DC: National Academies Press (2025).

[18] Du X Xu J Zhang S Liu L. Diagnostic value of platelet to high-density lipoprotein cholesterol ratio in abdominal aortic aneurysms. Front Cardiovasc Med. (2025) 12:1687265. 10.3389/fcvm.2025.1687265 41246026 PMC12611948

[19] Chen J Wang B Liu C Meng T Zhou Y. Association between platelet to high-density lipoprotein cholesterol ratio (PHR) and hypertension: evidence from NHANES 2005-2018. Lipids Health Dis. (2024) 23(1):346. 10.1186/s12944-024-02342-3 39462374 PMC11514891

[20] Çiçek G Açıkgöz SK Yayla Ç Kundi H İleri M. White blood cell count to mean platelet volume ratio: a novel and promising prognostic marker for ST-segment elevation myocardial infarction. Cardiol J. (2016) 23(3):225–35. 10.5603/CJ.a2016.0001 26779969

[21] Demir G Karaca G Ekmekci A Safaei S Kimiaei A Emre A. White blood cell/mean platelet volume ratio as a predictor of long-term outcomes but not coronary artery disease severity in non-ST elevation myocardial infarction patients. Cureus. (2023) 15(12):e51423. 10.7759/cureus.51423 38299134 PMC10828626

[22] Thomas KS Puthooran DM Edpuganti S Reddem AL Jose A Akula SSM. Reperfusion injury in STEMI: a double-edged sword. Egypt Heart J. (2025) 77(1):83. 10.1186/s43044-025-00683-7 40911117 PMC12413384

[23] Arrivi A Barillà F Carnevale R Sordi M Pucci G Tanzilli G. Protective biomolecular mechanisms of glutathione sodium salt in ischemia-reperfusion injury in patients with acute coronary syndrome-ST-elevation myocardial infarction. Cells. (2022) 11(24):3964. 10.3390/cells11243964 36552727 PMC9777519

[24] Offermanns S. Activation of platelet function through G protein-coupled receptors. Circ Res. (2006) 99(12):1293–304. 10.1161/01.RES.0000251742.71301.16 17158345

[25] Henes J Friedel M Stübenrath R Laspa Z Dicenta-Baunach V Rohlfing A-K. Platelet subfractions significantly differ in STEMI patients depending on angiographic findings. Clin Res Cardiol. (2025) 114(1). 10.1007/s00392-025-02625-438565712

[26] von Hundelshausen P Schmitt MM. Platelets and their chemokines in atherosclerosis-clinical applications. Front Physiol. (2014) 5:294. 10.3389/fphys.2014.00294 25152735 PMC4126210

[27] Schanze N Hamad MA Nührenberg TG Bode C Duerschmied D. Platelets in myocardial ischemia/reperfusion injury. Hamostaseologie. (2023) 43(2):110–21. 10.1055/a-1739-9351 35913081 PMC10132858

[28] Yang HT Liu JK Jiang ZH Yang Y Zhang J. Association of HDL-C levels with all-cause mortality in ACS patients after PCI: a multicenter prospective cohort study. Heart Vessels. (2025) 40(12):1–10. 10.1007/s00380-025-02568-w 40553120

[29] Das P Ingole N. Lipoproteins and their effects on the cardiovascular system. Cureus. (2023) 15(11):e48865. 10.7759/cureus.48865 38106760 PMC10724412

[30] Rached F Lhomme M Camont L Gomes F Dauteuille C Robillard P. Defective functionality of small, dense HDL3 subpopulations in ST segment elevation myocardial infarction: relevance of enrichment in lysophosphatidylcholine, phosphatidic acid and serum amyloid A. Biochim Biophys Acta. (2015) 1851(9):1254–61. 10.1016/j.bbalip.2015.05.007 26037829

[31] Sbrana F Dal Pino B Emdin M. Impaired HDL cholesterol function and high interleukin-1β levels hold prognostic value after ST-elevation myocardial infarction. Eur J Prev Cardiol. (2025) 32:1286–7. 10.1093/eurjpc/zwaf040 39870580

[32] Zheng Y. The study on relationship between platelet and coronary heart disease. Hebei Med J. (2000) (5):331–2.

[33] Xin M Hongyan B Qian L Ning Z. Correlation between fibrinogen to albumin ratio, platelet parameters and coronary artery lesion degree. Hebei Med. (2025) 31(8):1364–9. 10.3969/j.issn.1006-6233.2025.08.024

[34] Zhang H Xu Y Xu Y. The association of the platelet/high-density lipoprotein cholesterol ratio with self-reported stroke and cardiovascular mortality: a population-based observational study. Lipids Health Dis. (2024) 23(1):121. 10.1186/s12944-024-02115-y 38659020 PMC11040779

[35] Jialal I Jialal G Adams-Huet B. The platelet to high density lipoprotein -cholesterol ratio is a valid biomarker of nascent metabolic syndrome. Diabetes Metab Res Rev. (2021) 37(6):e3403. 10.1002/dmrr.3403 32886844

[36] Li J Chen L Xiao L You Q Luo N Shi J. The U-shape relationship between platelet/high-density lipoprotein cholesterol ratio and 1-year all-cause mortality in acute coronary syndrome patients: a retrospective cohort study. Eur J Med Res. (2026) 31. 10.1186/s40001-026-04235-w

[37] Shimabukuro M. Leucocyte count: inflammatory and ROS biomarkers of ASCVD. J Atheroscler Thromb. (2024) 31(6):861–3. 10.5551/jat.ED258 38599821 PMC11150727

[38] Sadowski M Zabczyk M Undas A. Coronary thrombus composition: links with inflammation, platelet and endothelial markers. Atherosclerosis. (2014) 237(2):555–61. 10.1016/j.atherosclerosis.2014.10.020 25463088

[39] Weng Y Gao Y Zhao M Zeng T Huang J Xie H. The white blood cell count to mean platelet volume ratio for ischemic stroke patients after intravenous thrombolysis. Front Immunol. (2022) 13:995911. 10.3389/fimmu.2022.995911 36263052 PMC9574706

